# Improving Quality of Multidisciplinary Team Meetings in Our Community Mental Health Team in NHS Greater Glasgow and Clyde, Scotland

**DOI:** 10.1192/bjo.2024.340

**Published:** 2024-08-01

**Authors:** Prerna Ambardar, Rachel Brown

**Affiliations:** GGC, Glasgow, United Kingdom

## Abstract

**Aims:**

To ensure smooth running of Multidisciplinary team (MDT) in Community mental health team (CMHT) and reviewing MDT structure for better functioning at Parkview Mental health Resource centre.

On a Friday two Multidisciplinary teams (MDTs) were running online on Microsoft teams simultaneously. The same staff was running the two MDTs, so staff input could be limited at times and staff would dip in and out of MDTs. Discussion around ways of improving this so that both MDTs run smoothly. Also, there was no formal structure to MDT meetings. It was decided that improvement in Quality of MDT needs to be addressed.

**Methods:**

Initially numerous discussions held online with Parkview team, nursing colleagues.

CMHT Quality improvement group was set up and a meeting was arranged where everyone's ideas were considered.

A pilot project was first introduced in March 2022 and audited in July 2022. Plan, do, study, act (PDSA) cycle was carried out.

Plan

Two nursing teams to be setup which will feed back into the two MDTs on alternate weeks. This will reduce nursing teams having to come in and out of one MDT to join other MDT, hence increasing the efficacy of the MDT.

Devise a new template to provide formal structure for the MDT presentation.

Do

Trial the new setup of two nursing teams.

Study

Ask all MDT staff members for feedback on the working of MDT.

Act

Reformat the Structured template and distribute to all staff members.

**Results:**

100% staff felt that new structure of MDT was useful.

84% staff satisfied with the new way of running of MDT.

84% staff satisfied with having designated teams for MDT.

**Conclusion:**

Having Designated MDT teams and a structured format helped in robust functioning of the MDT in the CMHT.

